# EHMTI-0065. Effects of antiepileptic drugs on spreading depression in the chick retina: implications for migraine prophylaxis

**DOI:** 10.1186/1129-2377-15-S1-G23

**Published:** 2014-09-18

**Authors:** J Mascarenhas de Moraes, J Houzel, L Cavalcanti

**Affiliations:** 1 Neurology, Federal University of Rio de Janeiro (UFRJ), Rio de Janeiro, Brazil; 2 Biophysics, Federal University of Rio de Janeiro (UFRJ), Rio de Janeiro, Brazil

## 

Spreading Depression (SD) is an answer of the nervous tissue to a different type of local stimulus. The knolled of this phenomenon is fundamental for the correct treatment migraine .

We analyze the effects of antiepileptic drugs, on the spreading depression (SD) in isolated retina of chick’. We studied five drugs with proven effect on GABAergic transmission: Topiramate, Valproate semisodium, Gabapentin, Lamotrigine and Levetiracetam. Chicks´ retinas were kept in superfusion chamber, with Ringer’s reference solution. the amplitude, the deflagration threshold (after chemical stimulus with KCl-) and the absolute refractory period of the SD, with and without the drugs were analyzed. Subsequently, the speed and amplitude parameters, also with and without the drugs, were analyzed in vivo . In addition, the GABA-transaminase enzyme activity was determined. Analysis of variance was used to determine the activity of GABA-transaminase.

We verified that all the drugs, particularly Topiramate reduce the speed and amplitude in a dose-dependent and reversed manner, in vitro as well as in vivo. All the drugs also increase, in a reversible form, the deflagration threshold for the SD, after chemical stimulus with KC-, in specific concentrations. It was also verified, that all the drugs increase, in a reversible form, the absolute refractory period. Topiramate was considered the most effective drug. The enzyme GABA-transaminase displayed slight decrease activity.

These results reinforce the notion that SD is a subjacent and relevant factor for the pathophysiology of migraine .the treatment of this pathology must emphasizes the use of antiepileptic drugs, in special Topiramate.

No conflict of interest.

